# Carbon-Assisted Q-Switched Nd:YAG Laser and Microneedling Delivery of Botulinum Toxin: A Prospective Pilot Study

**DOI:** 10.1097/PRS.0000000000011198

**Published:** 2023-11-14

**Authors:** Piergiorgio Turco, Claudio Conforti, Francesco D’Andrea, Simone La Padula

**Affiliations:** Napoli, Trieste, and Rome, Italy; and Créteil, France; From the 1 Department of Plastic and Reconstructive Surgery, Università degli Studi di Napoli Federico II; 2 Dermatology Clinic, University of Trieste, Maggiore Hospital; 3 IDI-IRCCS, Dermatological Research Hospital; 4 Department of Plastic, Reconstructive and Maxillofacial Surgery, Henri Mondor Hospital, University Paris XII.

## Abstract

**Background:**

Carbon-assisted, Q-switched, neodymium-doped yttrium-aluminum-garnet laser treatment consists of applying a topical carbon suspension all over the face, followed by irradiation with a Q-switched 1064-nm neodymium-doped yttrium-aluminum-garnet laser. The delivery of multiple microdroplets of dilute onabotulinum toxin type A into the dermis has been investigated as a tool for facial rejuvenation. The aim of this study was to assess the effectiveness of the combined treatment with botulinum toxin and carbon peel laser (performed with a standardized technique) in patients with seborrhea, dilated pores, and wrinkles, and to demonstrate its benefits in improving the overall skin aspect.

**Methods:**

Patients enrolled in this prospective pilot study underwent 3 sessions of the combined treatment carried out 3 months apart. To evaluate the improvement of skin texture, wrinkles, dilated pores, and acne lesions, the Fitzpatrick Wrinkle Assessment Scale, the Physician Global Aesthetic Improvement Scale, a photographic scale for the pore assessment, and the Investigator Global Assessment of Acne scale were used. The FACE-Q was also administered to assess patient satisfaction. The scores obtained were compared using a paired *t* test.

**Results:**

Twenty patients were recruited. The differences between pretreatment and posttreatment scores were statistically significant (*P* < 0.05) on the Fitzpatrick Wrinkle Assessment Scale, Physician Global Aesthetic Improvement Scale, Investigator Global Assessment of Acne scale, FACE-Q, and photographic scale for the pore assessment.

**Conclusions:**

This combined protocol could be considered as a useful tool to treat skin flaws that affect texture, microroughness, and seborrhea and to reduce the size of enlarged pores. Its versatility allows for customized treatment with minimal discomfort to patients.

**CLINICAL QUESTION/LEVEL OF EVIDENCE:**

Therapeutic, IV.

Carbon-assisted, Q-switched, neodymium-doped yttrium-aluminum-garnet (Nd:YAG) laser treatment has been used to improve skin texture, superficial wrinkles, acne lesions, and dilated pores. This technique consists of applying a topical carbon suspension all over the face for approximately 10 minutes, followed by irradiation with a Q-switched 1064-nm Nd:YAG laser. The irradiation eliminates small carbon molecules binding both the corneocytes and serum within the hair follicles; the high temperature generated reduces sebum production by sebaceous glands and inhibits *Cutibacterium acnes* replication.^1^

Botulinum toxin type A (BTX-A) injection is one of the most popular noninvasive cosmetic procedures performed worldwide. The injection of multiple microdroplets of diluted BTX-A into the dermis, referred to as “micro-Botox,”^2^ has been investigated as a tool for facial rejuvenation. Several studies have demonstrated the benefit of BTX-A in the treatment of facial erythema and improving skin texture.^3,4^

Calvani et al.^5^ demonstrated the superficial injection needling botulinum technique with dermal injection of microdoses of botulin toxin, not using a traditional syringe but with a needling technique that consists of multiple microdroplets delivered by an electrical device. The aims are to decrease sweat and sebaceous gland activity to improve skin texture and sheen and to target the superficial layer of muscles that find attachment to the undersurface of the dermis, causing visible rhytides. These methods were described individually in the past, but scientific data supporting their efficacy and safety have only recently been reported in small case series, even if they are sometimes controversial. The aim of this study was to assess the effectiveness of the combined treatment with botulinum toxin and carbon peel laser (performed with a standardized technique) in patients with seborrhea, dilated pores, and wrinkles, and to demonstrate its benefits in improving the overall appearance of the skin.

## PATIENTS AND METHODS

Only patients older than 18 years and affected by mild or moderate acne with poor response to conventional treatments (antibiotics with or without retinoids), enlarged pores of the face, and fine wrinkles were enrolled in this prospective pilot study. Exclusion criteria were oral isotretinoin treatment, an ongoing autoimmune skin disease, pregnancy, breastfeeding, and absence of informed consent. Patients enrolled in this study had Fitzpatrick skin types II through IV. The study design was approved by the ethics committee of our institution. All procedures in the study involving human participants were performed in accordance with the ethical standards of institutional and/or national research committees and with the 1964 Declaration of Helsinki and its later amendments or comparable ethical standards. Ethical approval was given by the French institutional committee (reference no. 2022-A231481-97).

All patients underwent 3 sessions of carbon-assisted Q-switched Nd:YAG laser and microneedling delivery of botulinum toxin, carried out 3 months apart. After each patient’s skin was cleaned (0.05% sodium hypochlorite electrolytic solution), a carbon topical solution (NaturaPeel; Quanta System, Deka, Italy) was applied to the face for 10 minutes. After that, a nanosecond Q-switched laser (KJU Lasering Group, Modena, Italy) was used, with a 5-mm spot, a 550-mJ/cm^2^ fluence, and a 5-Hz frequency. After the laser treatment was performed, the skin was cleaned again (0.05% sodium hypochlorite electrolytic solution) to remove any possible carbon residue.

Onabotulinum toxin type A (Vistabex; Allergan), in lyophilized form, was resuspended in saline solution (0.7 to 0.8 mL) for injection (0.9% sodium chloride). Resuspensions were made by reducing the amounts suggested by the manufacturers, according to dilutions based on personal experience and in accordance with previous study,^5^ as shown in Table 1.

**Table 1.** Dilutions of Onabotulinum Toxin A Used in This Study

Botulinum Toxin	Standard Resuspension	Proposed Resuspension	Modified Resuspension (>65 Yr)
Onabotulinum toxin type A (50 IU)	1.25 mL of 0.9% NaCl	0.80 mL of 0.9% NaCl	0.70 mL of 0.9% NaCl

NaCl, sodium chloride.

After disinfection, patients’ faces were washed with sterile gauze soaked in 0.9% sterile saline. After drying, the area to be treated in the session was delineated.

The treated areas and the amount of toxin used were standardized. Only the facial area was treated, separate from the neck and jaw, and a grid was created based on the tip diameter of the medical device used (m.pen; Mesoestetic, Barcelona, Spain).

**Fig. 1.**
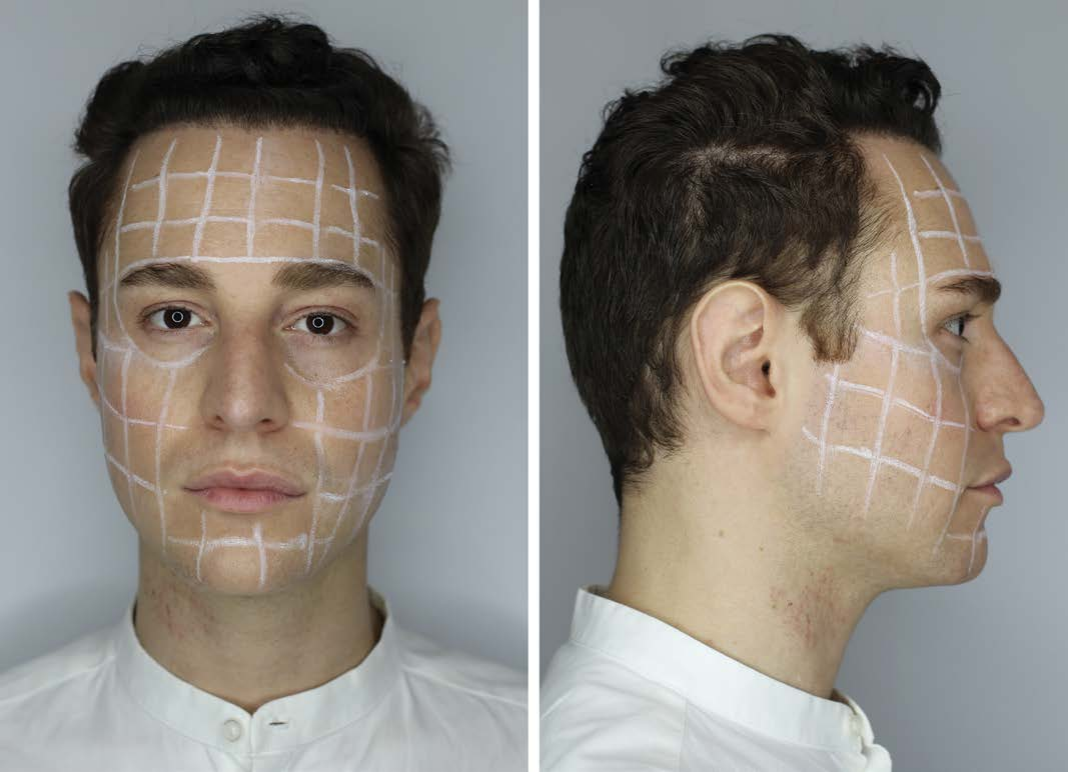
Face division grid. The grid is drawn according to the size of the head of the microneedling device, which in this case had a diameter of 1 cm.

**Fig. 2.**
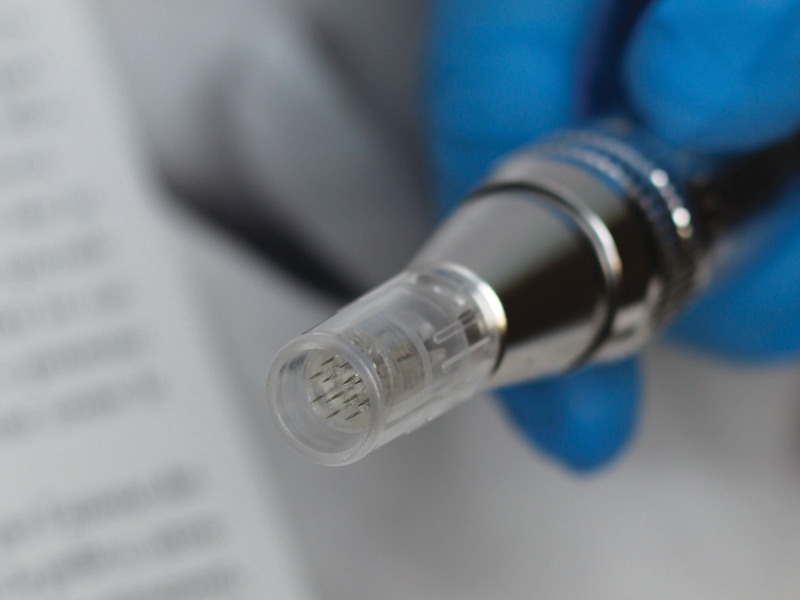
Close-up view of the tip of the microneedling device used in this study.

As previously stated by Calvani et al.,^5^ it is possible to divide the treated area into quadrants (in this case, the amount of recombined toxin in the physiologic solution is divided by the number of quadrants) or to divide the face area by building a grid. Each grid unit must have the size of the head of the medical device used: the head of the instrument used in this study had a diameter of 1 cm (Fig. 1). The microneedling device used was set with a 12-needle head with a depth of 1.5 mm extended to 2.0 mm (Fig. 2).

The maximum amount of toxin used in each patient was 50 U (25 U per side) recombined in 0.7 to 0.8 mL of saline. This amount was injected homogeneously across the surface marked by the grid to evenly treat the patient’s entire face.

To better dose the drops, a 0.5-mL syringe with a 32-gauge needle was used. This allowed for the formation of very small drops, because the thin needle and the easy-to-maneuver syringe allowed for a homogeneous distribution.

The technique involved treating, in a single session, the areas selected by administering a drop of resuspended botulinum (10 μL) on the skin and immediately placing the microneedling device firmly above the liquid on the same spot for 3 seconds to deliver the solution intradermally. (See Video [online], which demonstrates the microneedling delivery of botulinum toxin; the video has been sped up.) Approximately 3000 perforations per area were applied (the medical device performs approximately 10,000 perforations/minute), and the depth of penetration of the microneedles was set at 2 mm.

**Video.** This video demonstrates the microneedling delivery of botulinum toxin; the video has been sped up.

Moderate pressure was applied with the device on the skin area. On bony prominences, it was necessary to pinch the skin with the fingers to lift it up from the underlying bone. This procedure was repeated throughout the area outlined until the end of the treatment.

The factors that were essential to penetration of the greatest amount of botulinum toxin into the tissue for the desired effect were as follows: the use of moderate pressure, which helped to increase the depth of the tip; the adopted dispenser, which consisted of a 0.5-mL syringe and a 30-gauge needle to allow for the formation of small drops; and a 3-second needle application time, which helped to increase the microtrauma, perforation, and intake of toxin. All patients underwent home disinfection of the treated areas for the first night with application of 0.05% sodium hypochlorite electrolytic solution. Patients washed their face twice a day with a specific facial cleanser (Acnever AHA/BHA Purifying Cleanser; Miamo, Milan, Italy), applied a sunscreen gel daily (Heliocare 360° Water Gel SPF 50+; Difa Cooper, Cantabria Labs Group, Milan, Italy), and used a moisturizing lotion (Acnever Cream; Miamo) before going to bed for the duration of the protocol.

Digital photographs were taken before, 1 month after, and 3 months after the procedure in standard light conditions. All patients were photographed in both frontal and profile views using the same digital camera (Canon EOS 1300D) with standardized settings. Photographs were shown to 3 independent assessors (2 plastic surgeons and 1 dermatologist) for the evaluation of the results.

To assess the improvement of thin wrinkles (because stimulation of the dermis induced by laser-irradiated carbon shock waves can result in improvement of wrinkles), the Fitzpatrick Wrinkle Assessment Scale was used for the assessment of thin/deep lines on the face (0 = absence of wrinkles, 6 = deep and diffuse wrinkles).^6^ The Physician Global Aesthetic Improvement Scale was used to express overall improvement (1 = exceptional improvement, 5 = worsened patient).^7^ A photographic scale for the pore assessment (1 = no obvious pores , 6 = enlarged pores)^8^ and the Investigator Global Assessment of Acne (IGA) for the classification of acne lesions (0 = no inflammatory lesions, 3 = many comedones, inflammatory lesions, and nodules)^9^ were also used to complete the evaluation of our treatment.

The FACE-Q was also administered to assess patients’ satisfaction before the first session and 3 months after the last session. Rasch measurement theory analysis was used to evaluate reliability and validity of evaluations.

The raters gave their score before and 3 months after treatment. The scores, following a normal distribution obtained by each rater, were compared using a paired *t* test. These assessments were performed 3 months after the third treatment session, comparing these scores to the baseline. A value of *P* < 0.05 was considered as a cutoff for significance.

To compare categorical data, we used the chi-square and Fisher exact tests, considering results significant at *P* = 0.05. All analyses were performed using Prism version 5 software (GraphPad, Boston, MA). All authors had full access to the data and took full responsibility for the integrity of the data.

## RESULTS

A prospective pilot study was performed including patients treated from January of 2020 to December of 2022 in the context of a private practice. Twenty patients (7 women and 13 men) aged between 20 and 68 years were recruited (mean age ± SD, 34.1 ± 14.78 years). Baseline characteristics of patients are reported in Table 2. Informed consent was obtained from all study participants.

**Table 2.** Characteristics of Patients Treated with the Combined Protocol (*n* = 20 Patients)

Baseline Characteristics	No. (%)
Sex	
Female	7 (35)
Male	13 (65)
Mean age, yr	
Female patients	33.21 ± 12.8
Male patients	33.57 ± 11.9
*P*	0.4
Phototype	
1	0
2	4 (20)
3	11 (55)
4	5 (25)

**Table 3.** Characteristics of Patients Treated with the Combined Protocol (*n* = 20 Patients)

	Before Treatment (%)	After Treatment (%)	*P * ^a^
FWAS			0.000349
1	3 (15)	7 (35)	
2	11 (55)	8 (40)	
3	5 (25)	5 (25)	
4	1 (5)	0	
Pores			<0.00001
No obvious pores	0	7 (35)	
Minimal pores	2 (10)	12 (60)	
Mild pores	12 (60)	1 (5)	
Moderate pores	6 (30)	0	
IGA			<0.00001
No inflammatory and noninflammatory lesions	0	5 (25)	
Few comedones and <1 small inflammatory lesion	8 (40)	12 (60)	
Dozens of comedones and several inflammatory lesions	12 (60)	12 (60)	
Many comedones, several inflammatory lesions, and <1 nodule	0	0	
GAIS			
Exceptional improvement		2 (10)	
Very improved		10 (50)	
Improved		8 (40)	
Unaltered			
Worsened			

FWAS, Fitzpatrick Wrinkle Assessment Scale; GAIS, Global Assessment Investigator Scale.

a *P* according to *t* test for paired samples for continuous variables, and according to χ^2^ and Fisher exact tests for categorical data.

**Table 4.** Characteristics of Patients Treated with the Combined Protocol (*n* = 20 Patients)

FACE-Q (Rasch)	Men	Women	*P * ^a^
Mean score			<0.00001
Before treatment	30.02 ± 3.2	30.1 ± 2.7	0.3
After treatment	76.3 ± 1	75.8 ± 2.4	0.2

a *P* according to *t* test for paired samples for continuous variables, and according to χ^2^ and Fisher exact tests for categorical data.

The treatment did not produce ecchymosis requiring cosmetic camouflage. No severe adverse reactions or complications (eg, postinflammatory hyperpigmentation and hypopigmentation, infection, allergic reactions, thermal damage, unsatisfactory results) were reported. All patients reported mild transient erythema that disappeared 1 to 3 hours later. Five patients (25%) reported mild dryness and desquamation; however, these findings resolved spontaneously within a few days.

The difference between pretreatment and posttreatment scores on the Fitzpatrick Wrinkle Assessment Scale were statistically significant (*P* = 0.000349), demonstrating an improvement of the thin and deep lines on the face. The Physician Global Aesthetic Improvement Scale showed a very good overall improvement (2.2 ± 0.65) in the appearance of the face. A significant difference between pretreatment and posttreatment scores on the photographic scale for the pore assessment was observed (*P* < 0.00001).

**Fig. 3.**
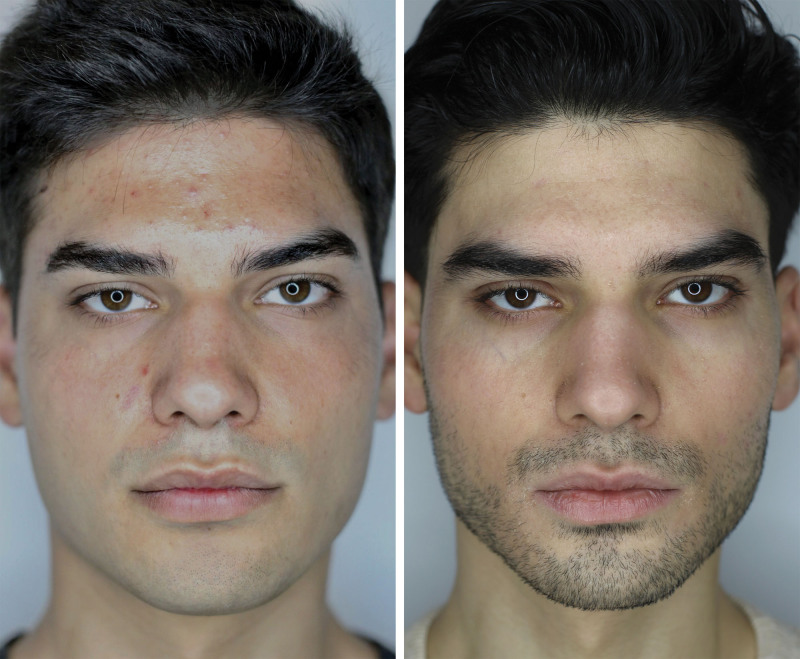
Clinical evaluation of a 22-year-old male patient in response to the combined protocol. Representative photographs were taken at baseline (*left*) and 3 months after the end of the protocol (*right*).

IGA scores for the classification of acne lesions improved significantly after treatment (*P* < 0.00001) (Fig. 3). The patients were very satisfied with the treatment, with a statistically significant difference between pretreatment and posttreatment FACE-Q scores (*P* < 0.00001). A global improvement was observed in all patients enrolled in this study; no inflammatory acne lesion was treated directly with the microneedling device to avoid skin infections (only perilesional skin was treated).

**Fig. 4.**
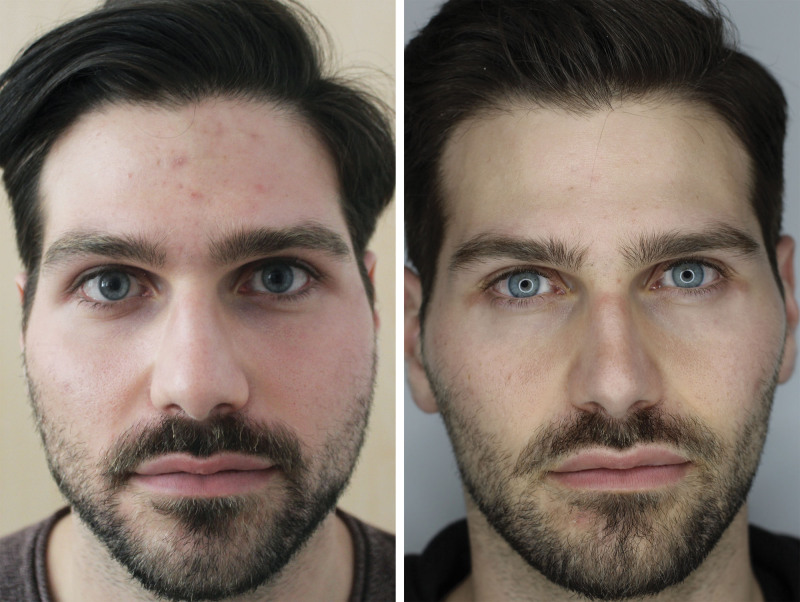
Clinical evaluation of a 25-year-old male patient in response to the combined protocol. Representative photographs were taken at baseline (*left*) and 3 months after the end of the protocol (*right*).

In particular, a significant improvement in the clinical aspect of patients’ pores and acne lesions was observed, as confirmed by the IGA scores and the photographic scale for the pore assessment (Fig. 4). (**See Figure, Supplemental Digital Content 1**, which demonstrates the clinical evaluation of a 24-year-old female patient in response to the combined protocol; photographs were taken at baseline and 3 months after the end of the protocol, http://links.lww.com/PRS/G913.)

## DISCUSSION

The results of this study suggest that the combined protocol presented in this article could be used to improve irregular skin texture, microroughness, and seborrhea, and to reduce the size of enlarged pores, because of the combined action of the microneedling delivery of botulinum toxin and carbon-assisted Q-switched Nd:YAG laser irradiation. Nowadays, the aesthetic use of BTX-A is supported by a broad literature base.^10–15^ Although the traditional injection points and the standardized dosage are still commonly used,^1,10^ most surgeons have changed their approach to tailor dosage and injection points on an individual basis. This includes taking into account differences in muscle anatomy, muscle strength, baseline asymmetries, and relationships to other aesthetic units, along with the patient’s desired outcome. The most common use of botulinum neurotoxin in aesthetic medicine is to treat glabellar, forehead, and periorbital lines, but other cosmetic applications of neuromodulators to the middle and lower face^11^ can lead to interesting aesthetic improvement.

As previously published, there are several factors that determine sebum production and pore size: the activity of the arrector pili muscle and the acetylcholine-mediated activation of local muscarinic receptors in the pilosebaceous unit are strongly related.^11,16^ Even if the role of the nervous system and acetylcholine in sebum production is not well known, the improvement in skin condition, both self-assessed and as measured by medical scores, is significant.^11,17^

The injection of multiple microdroplets of dilute BTX-A into the dermis, referred to as micro-Botox, a term first used by Wu,^2^ has been investigated as a tool for facial rejuvenation. Several studies have demonstrated the benefit of BTX-A in treating facial erythema and improving skin texture.^3,4^

Wu^2^ first described this technique and applied it only to the lower face and neck; however, to perform our technique, we applied a layer of ELA-max (5%) over the area to be injected 20 minutes before the intradermal injections and then washed it off. Further, we added an additional 0.5 mL of lidocaine (0.5%) with or without adrenaline to our injectable solution.

The micro-Botox described by Wu^2^ is characterized by a greater dilution of type A neurotoxin and by the fact that the injections are performed intradermally, using 30 gauge, 4- to 12-mm needles.

Although there are no data in the literature on variation of full effect or duration of the toxin attributable to the added injectable anesthetic, in this study, only the saline solution was used for botulinum reconstitution. Moreover, adrenaline may leave multiple points of blanching, and some patients will dislike this temporary speckled appearance.

Our procedure, however, does not require an anesthetic cream to be used before the injections or injectable anesthetic to be added in the resuspended solution, because the delivery of the neurotoxin through microneedling is significantly less painful. No interruptions of treatment were reported (because of pain issues), and the compliance of patients involved in the study was remarkable.

Wanitphakdeedecha et al.^18^ described the beneficial effect of intradermal microdosing of abobotulinumtoxinA for face lifting. Their results in terms of the duration of the effect are comparable to our data.

Sayed et al.^4^ reported the beneficial effect of intradermal injections of botulinum toxin in managing enlarged facial pores and seborrhea in a cohort of 20 patients. Their study showed that this procedure was both effective and safe in managing excess sebum and facial pores, with acceptable results lasting for an average period of 4 months. Our results are comparable to theirs, particularly in terms of the duration of the injection’s effectiveness.

In addition to traditional syringe injection, BTX-A can also be administered by means of multiple microdroplets using an electrical device. Previous studies on this topic have been published in the literature.^5,19^

Calvani et al.^5^ described their personal superficial injection needling botulinum technique of microinfiltration with botulin toxin applied to 63 patients to decrease sweat and sebaceous gland activity, to improve skin texture and sheen, and to target the superficial layer of muscles that find attachment to the undersurface of the dermis, causing visible rhytides. The technique adopted in our study and also by Calvani et al.,^5^ unlike micro-Botox, contemplates a hyperconcentration and not a hyperdilution. Moreover, the inoculation technique is different because no infiltration needles are used (microneedling is used instead of the classic syringe).

Irregular skin texture, dilated facial pores, and seborrhea are very common skin flaws that may benefit from a combined treatment with lasers to obtain a better improvement. The 1064-nm Q-switched Nd:YAG laser has been used in nonablative skin rejuvenation. It can deliver energy to the dermis, and the heat-induced damage results in collagen and elastin stimulation.

Roh et al.^16^ studied the role of Q-switched Nd:YAG laser in patients with dilated pores in a split-face study. The treated side showed significant improvement compared with the control side (*P* < 0.05), and the results were maintained until 8 weeks after the last session.

In another split-face study, Chung et al.^20^ compared the use of Q-switched Nd:YAG laser alone versus its combination with carbon solution; clinical improvement was noticed in 75% of studied cases after carbon application versus 67% without carbon. They supposed that the particles of the carbon solution on the stratum corneum are blocked in the dilated pores and act as an exogenous chromophore that absorbs Nd:YAG laser light. After absorption of laser energy, the carbon particles are heated very fast and explode. This generates kinetic energy that destroys the stratum corneum and the pore walls, and results eventually in the reduction of pore size and sebum production.

Lee et al.^21^ used a Q-switched Nd:YAG laser with carbon solution in 24 patients with dilated pores. They reported a significant improvement in pore size and sebum production and an improvement in the skin texture and tone (*P* < 0.001) in all patients after 4 sessions. Eldeeb et al.^22^ used a 1064-nm Q-switched Nd:YAG laser, combined with topically applied carbon solution, for the treatment of wide facial pores. After the end of treatment, a marked to excellent response (51% to 100% improvement of their wide pores) was obtained in 75% of the studied subjects, and the results were maintained in 25% of the patients at the 6-month follow-up visit.

Conforti et al.^1^ analyzed with validated scales and medical scores the beneficial effect of the treatment of skin texture, dilated pores, and acne lesions with the Q-switched 1064-nm Nd:YAG laser combined with a topically applied carbon. However, to the best of our knowledge, this is the first study that analyzed the beneficial effect of a combined protocol of botulinum inoculation and Q-switched 1064-nm Nd:YAG laser technique on skin quality by means of instrumental and, thus, objective assessment.

Based on these observations, we chose to develop a combined treatment approach. The evidence we gathered provides strong support for the rationale underlying the combined treatment used in our study.

### Strength and Limitations

The primary limitations of this study include small sample size and the absence of control and/or comparative groups (patients were treated only with either. microneedling delivery of botulinum toxin or the Q-switched Nd:YAG laser). However, the strengths of the study are its prospective nature; the use of several medical scales to obtain objective evaluation; the representativeness of the sample (even if small), which allows for evaluation of the efficacy of the treatment; and the fact that this is the first study in the literature that describes a complete standardized and combined protocol (these methods were described only singularly in the past).

## CONCLUSIONS

The results of this prospective pilot study suggest that this combined protocol could be considered as a useful tool to treat skin flaws affecting texture, microroughness, and seborrhea, and to reduce enlarged pore size. Its high versatility allows for customized treatment with minimal discomfort and high tolerability. Further studies are needed to determine the effect of this combined treatment in a larger cohort of patients and to assess the efficacy after a longer follow-up period.

## DISCLOSURE

The authors have no financial interest to declare in relation to the content of this article.

## PATIENT CONSENT

Patients provided written informed consent for the use of their images.

## Supplementary Material


