# Supplementary figures and images for: Carbon-Assisted Q-Switched Nd:YAG Laser and Microneedling Delivery of Botulinum Toxin: A Prospective Pilot Study
Source: Plast Reconstr Surg. 2023 Nov 14;154(3):521–9. doi: 10.1097/PRS.0000000000011198 (PMC11346700; doi:10.1097/PRS.0000000000011198)

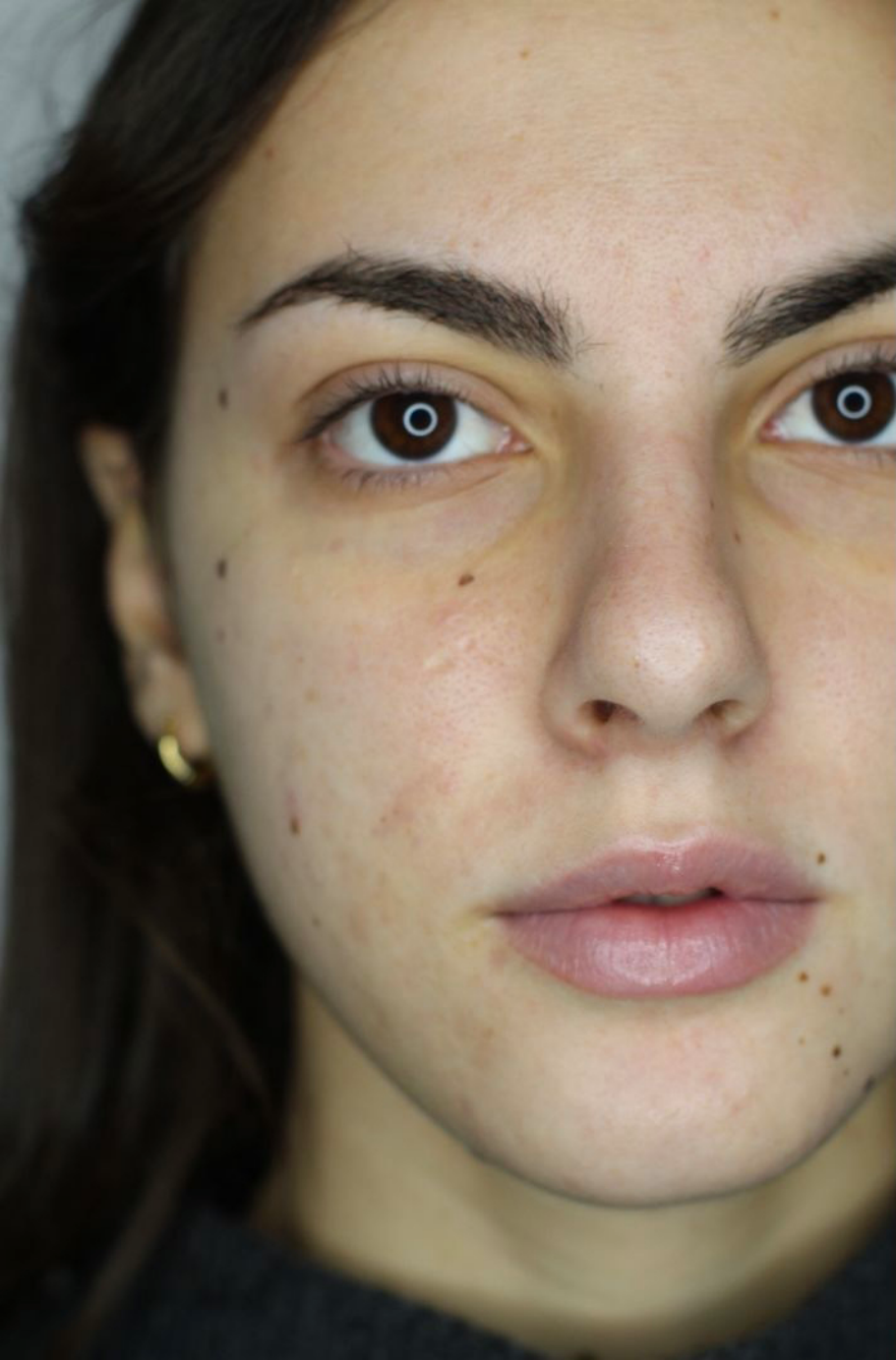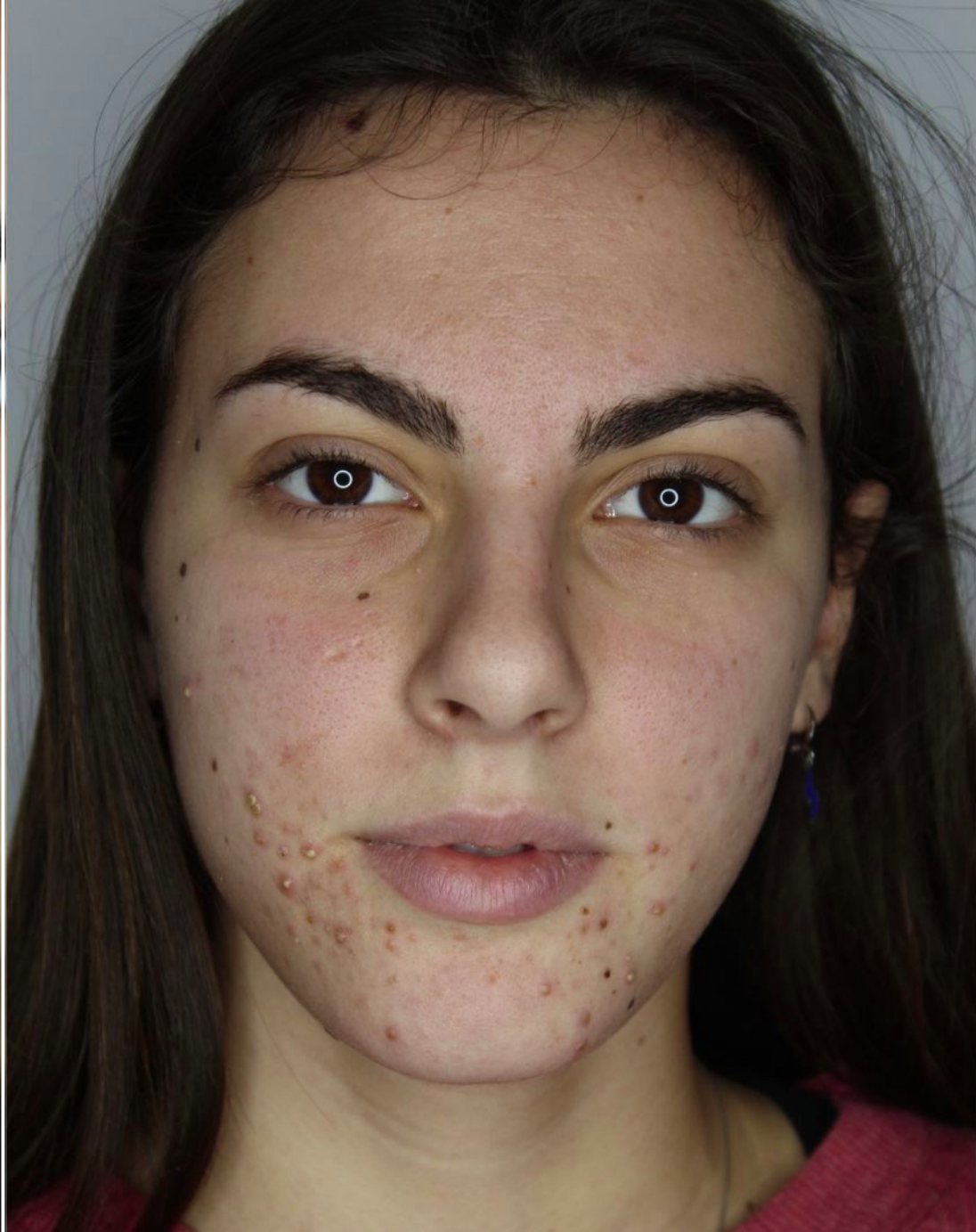

Supplement: Supplementary file 2 [file prs-154-0521-s002.pdf]
